# Frenulum Breve: An Addendum to Accessory Penile Frenulum

**DOI:** 10.1055/s-0045-1802628

**Published:** 2025-02-17

**Authors:** G. I. Nambi, C. Nanda Gopal

**Affiliations:** 1 Department of General Surgery, Nandha Medical College and Hospital, Erode, Tamil Nadu, India

**Fig. 1:**
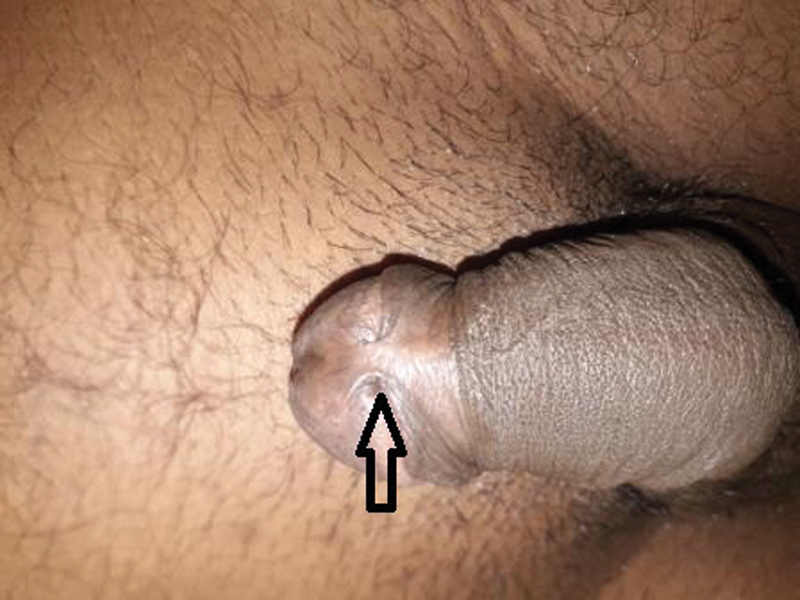
Short and wide penile frenulum.

Although an accessory penile frenulum 1 is a rare condition and may go unnoticed till death, there is another similar condition that may end up the same way. The condition is frenulum breve ( Fig. 1 ) or short penile frenulum, 2 3 which is the cause of painful erection, premature ejaculation, painful intercourse and bleeding from the penis due to rupture. Young males in their early 20s seeking circumcision for nonreligious reasons are the common patient group. This condition goes unnoticed by the patient and is not recognized by the patient till he seeks medical advice toward painful erection, painful intercourse, premature ejaculation, and bleeding from the penis after intercourse. Although circumcision relieves the patient from suffering, a simple frenuloplasty under local anesthesia gives equal relief and patient satisfaction.
